# Efecto de la férula oclusal en el dolor y el espacio articular en pacientes con trastornos temporomandibulares. estudio de serie de casos

**DOI:** 10.21142/2523-2754-1401-2026-280

**Published:** 2025-12-28

**Authors:** Jesús Giancarlo Pares-Ballasco, Kevin Fernando Huamaní-Bendezú, Juan Eduardo Vásquez-Izquierdo, Kenyie Jossuet Paucca-Calla, Javier Castro-Yanahida, Delia Mercedes Reynaldo-Padilla

**Affiliations:** 1 Universidad Nacional Mayor de San Marcos, Facultad de Odontología. Lima, Perú. jesus.pares@unmsm.edu.pe jcastroy@unmsm.edu.pe Universidad Nacional Mayor de San Marcos Universidad Nacional Mayor de San Marcos Facultad de Odontología Lima Peru jesus.pares@unmsm.edu.pe jcastroy@unmsm.edu.pe; 2 Hospital Nacional Dos de Mayo, Departamento de Odontoestomatología. Lima, Perú. kevin.huamani@upch.pe jeduardovasquez.i@gmail.com mechitadely08@gmail.com Hospital Nacional Dos de Mayo Departamento de Odontoestomatología Lima Perú kevin.huamani@upch.pe jeduardovasquez.i@gmail.com mechitadely08@gmail.com; 3 Universidad Norbert Wiener, Facultad de Odontología, Lima, Perú. kenyiepauccacalla@gmail.com Universidad Privada Norbert Wiener Universidad Norbert Wiener Facultad de Odontología Lima Peru kenyiepauccacalla@gmail.com

**Keywords:** férulas dentales, trastornos temporomandibulares, tomografía computarizada de haz cónico, Perú, occlusal splints, temporomandibular joint disorders, cone-beam computed tomography, Peru

## Abstract

**Introducción:**

Los trastornos temporomandibulares (TTM) afectan la articulación temporomandibular y los músculos masticatorios. La férula oclusal de estabilización (FOE) es un tratamiento convencional; sin embargo, la evidencia sobre sus efectos en el dolor percibido y el espacio articular temporomandibular es limitada.

**Objetivo:**

Describir los cambios en el dolor percibido y en los espacios articulares temporomandibulares en una serie de pacientes con TTM tratados con FOE. Métodos: Se incluyeron 13 pacientes con diagnóstico de TTM atendidos en un hospital. Se registró la intensidad del dolor mediante la escala visual análoga (EVA) antes y después de 30 días de uso continuo de la FOE. Mediante una tomografía computarizada de haz cónico en los cortes sagitales y coronales, se identificaron los signos degenerativos condilares y se midieron los espacios articulares (anterior, superior, posterior, medial y lateral) antes y después de la instalación de la FOE.

**Resultados:**

La media de edad fue 26,23 ± 13,94 años, y el 69,23 % fueron mujeres. La intensidad del dolor disminuyó significativamente de 6,15 ± 1,82 a 3,62 ± 2,06 (p < 0,001) y se observó que la reducción fue significativa únicamente en mujeres (p<0,05). El aplanamiento condilar se observó en el 69,23 % de los pacientes. Se evidenció una ligera disminución significativa en el espacio anterior derecho y un ligero aumento en los espacios anterior izquierdo, superior y medial derecho (p < 0,05).

**Conclusión:**

El uso de la FOE en pacientes con TTM evidenció una reducción significativa del dolor percibido y modificaciones inmediatas en los espacios articulares temporomandibulares.

## Abstract (en)

**Introduction:**

Temporomandibular disorders (TMD) affect the temporomandibular joint and masticatory muscles. The stabilization occlusal splint (SOS) is a conventional treatment; however, evidence regarding its effects on perceived pain and temporomandibular joint space is limited. Objective: To describe changes in perceived pain and temporomandibular joint spaces in a series of patients with TMD treated with SOS.

**Methods:**

Thirteen patients diagnosed with TMD and treated at a hospital were included. Pain intensity was recorded using a visual analog scale (VAS) before and after 30 days of continuous use of the FOE. Using cone beam computed tomography in sagittal and coronal sections, degenerative condylar signs were identified and joint spaces (anterior, superior, posterior, medial, and lateral) were measured before and after the installation of the SOS.

**Results:**

The mean age was 26.23 ± 13.94 years, with 69.23% being women. Pain intensity decreased significantly from 6.15 ± 1.82 to 3.62 ± 2.06 (p< 0.001), with the reduction being significant only in women (p<0.05). Condylar flattening was observed in 69.23% of patients. A slight but significant decrease was observed in the right anterior space and a slight increase in the left anterior, superior, and right medial spaces (p< 0.05).

**Conclusion:**

The use of SOS in patients with TMD showed a significant reduction in perceived pain and immediate changes in temporomandibular joint spaces.

## INTRODUCCIÓN

Los trastornos temporomandibulares (TTM) abarcan diversas complicaciones clínicas que afectan los músculos y las articulaciones de la región orofacial, y se caracterizan por dolor, ruidos articulares y disfunciones que pueden alterar los movimientos mandibulares o limitar la apertura bucal ^1^. Su etiología es multifactorial, ya que involucra factores biomecánicos, psicosociales y biológicos ^2^. El diagnóstico actual se realiza mediante el instrumento de examen de criterios diagnósticos para trastornos temporomandibulares (DC/TMD, por sus siglas en inglés), que comprende evaluación psicológica y tomografía computarizada de haz cónico (TCHC) ^3^. La intensidad del dolor percibido por el paciente suele evaluarse mediante la Escala Visual Análoga (EVA) ^4^.

El tratamiento de los TTM puede involucrar intervenciones quirúrgicas, no quirúrgicas o una combinación de ambas; sin embargo, menos del 10% de los pacientes con TTM requieren procedimientos quirúrgicos como la artroscopia o la cirugía abierta de la articulación temporomandibular (ATM) ^5^. Entre los tratamientos no quirúrgicos más utilizados se encuentran la fisioterapia, las férulas oclusales de estabilización (FOE) y la terapia farmacológica. No se han observado diferencias significativas entre estas opciones terapéuticas ^3^.

El uso de FOE en el tratamiento de pacientes con TTM ha demostrado ser eficaz en la reducción del dolor crónico percibido, así como en la mejora de la densidad ósea y la morfología del cóndilo ^6^^-^^8^. Estos beneficios se logran mediante la modificación de la relación topográfica entre el cóndilo y la fosa, lo que involucra la posición condilar y el espacio articular temporomandibular. Esta redistribución de las áreas de contacto entre las superficies articulares promueve una mejora en la función mandibular ^9^. No obstante, la evidencia disponible sobre el efecto de la FOE en el espacio articular temporomandibular sigue siendo limitada ^10^^,^^11^.

Durante tratamientos prolongados, de entre seis y doce meses, se han reportado modificaciones esqueléticas, dentoalveolares y de tejidos blandos, como el aumento de la altura facial inferior, la inclinación del plano mandibular y los planos oclusales, así como la disminución del *overbite*, el incremento del *overjet* y la retrusión del labio inferior ^12^^,^^13^. El seguimiento clínico riguroso y la planificación terapéutica individualizada son esenciales para optimizar los resultados funcionales y minimizar posibles alteraciones morfoestructurales. En este contexto, las FOE representan una alternativa terapéutica inicial de preferencia por su carácter no invasivo, reversible y de bajo costo, además que pueden combinarse con otras modalidades de tratamiento que potencian sus beneficios ^14^.

A pesar de las ventajas de las FOE y la mayor prevalencia del TTM en América del Sur (47%), en comparación con Asia (33%) y Europa (29%) ^15^, la literatura científica es escasa en el Perú y Latinoamérica con referencia al dolor percibido y el seguimiento imagenológico del espacio articular durante el tratamiento del TTM ^7^^,^^8^^,^^16^. En el Perú se ha reportado una elevada frecuencia de síntomas asociados con TTM en población adulta joven, predominantemente en mujeres, lo que evidencia la necesidad de investigaciones locales que profundicen en sus implicancias clínicas ^17^^,^^18^. 

En respuesta a esta necesidad, el presente estudio se realizó en el Hospital Nacional Dos de Mayo, institución de referencia que atiende a pacientes de diversas regiones del país, lo que aporta un contexto clínico relevante y puede generar evidencia preliminar para futuras investigaciones nacionales. Por lo tanto, el objetivo de este estudio fue describir los cambios en el dolor percibido y en los espacios articulares temporomandibulares en una serie de pacientes con TTM tratados con FOE.

## MÉTODOS

### Diseño del estudio y participantes

Se desarrolló una serie de casos conformada por 13 pacientes diagnosticados con TTM, atendidos en el Servicio del Adulto del Departamento de Odontoestomatología del Hospital Nacional Dos de Mayo, durante los meses de abril y mayo de 2024. Todos los pacientes presentaban dentición completa, requerían tratamiento mínimamente invasivo de la ATM y otorgaron su consentimiento informado para el seguimiento clínico. Se registraron variables clínicas como sexo, edad y tiempo transcurrido (en meses) desde el inicio de los síntomas del TTM hasta antes del tratamiento.

### Confección y características de la férula oclusal de estabilización

A cada paciente se le realizaron impresiones de las arcadas dentales superior e inferior con alginato cromático (Chromatic, Hygedent, China), además del registro oclusal con cera rosada (Cavex Set Up Regular, Cavex, Países Bajos). Los modelos de estudio fueron elaborados con yeso tipo IV (Fujirock, GC, Bélgica) y enviados a un técnico en prótesis dental para la confección en acrílico rígido de una FOE. 

Cada FOE instalada en boca presentaba guías caninas, soporte posterior y ligero contacto en el sector anterior. El procedimiento se realizó en dos citas clínicas: la primera destinada a la instalación y ajuste inicial de la FOE, y la segunda, a la verificación y realización de las modificaciones finales. Los ajustes oclusales se efectuaron con papel articular de 12 µm (Arti-Fol, Alemania) y pinzas Miller, hasta lograr una oclusión funcional. El dispositivo debía retirarse durante las comidas y el cepillado dental, y mantenerse un uso continuo durante el resto del tiempo. Cada paciente utilizó la FOE durante un periodo continuo de 30 días.

### Evaluación del dolor percibido

La intensidad del dolor fue evaluada mediante la escala visual análoga (EVA) ^4^, en la que 0 representa ausencia de dolor y 10 el dolor máximo imaginable en el día de la evaluación. Esta medición se realizó al inicio del tratamiento y luego de 30 días de uso de la FOE.

### Evaluación imagenológica

La evaluación de la ATM se realizó mediante TCHC. En la tomografía diagnóstica inicial se identificaron signos degenerativos condilares, como aplanamiento, erosión, osteofito, esclerosis y quiste subcondral, además de la valoración general del espacio articular. 

Para la medición de los espacios articulares se aplicó el método descrito por Januzzi *et al*. ^16^. En el corte coronal, desde la parte interior de la cabeza condilar mandibular, se trazó una línea horizontal entre los polos medial y lateral para definir el límite articular, una línea vertical por el centro condilar y dos líneas a 45° que delimitaron los espacios medial y lateral. En el corte sagital, se trazaron líneas de referencia para medir los espacios articular anterior, superior y posterior. Todas las mediciones se realizaron entre la cortical externa de la cabeza condilar mandibular y la cortical externa de la cavidad glenoidea del hueso temporal, de acuerdo con los trazos anatómicos de referencia.

La primera TCHC se efectuó como parte de la evaluación diagnóstica inicial y la segunda, tras la instalación y el ajuste clínico de la FOE. Todas las mediciones fueron realizadas por un especialista en radiología bucal y maxilofacial (Huamaní-Bendezú). El coeficiente de correlación intraclase fue 0,961 (IC95%: 0,920-0,981).

### Análisis de datos

Los datos fueron procesados con el programa estadístico Stata v.14.3. Se aplicó estadística descriptiva, y se incluyeron medidas de tendencia central (media y mediana) y dispersión (desviación estándar y rango intercuartílico). La normalidad de las variables se evaluó mediante la prueba de Shapiro-Wilk. Para explorar los cambios en el dolor percibido y los espacios articulares antes y después del uso de la FOE, se utilizó la prueba t de Student para muestras relacionadas en variables con distribución normal y la prueba de Wilcoxon para aquellas con distribución no paramétrica. Se adoptó un nivel de significancia estadística del 5% (p < 0,05).

## RESULTADOS

Se evaluaron 13 pacientes con diagnóstico de TTM. La edad media fue de 26,23 ± 13,94 años, y el 69,23% (n = 9) correspondió al sexo femenino. La mediana del tiempo transcurrido desde la aparición del dolor hasta antes del inicio del tratamiento fue de 12 meses (rango intercuartílico: 8 meses). En las TCHC de la ATM, se observaron 26 cóndilos y 26 espacios articulares temporomandibulares, de los cuales el 69,23% presentó aplanamiento condilar y el 46,15% evidenció disminución del espacio articular posterior (Tabla 1).

**Tabla 1:** Frecuencia de los hallazgos tomográficos observados en la articulación temporomandibular de pacientes con trastornos temporomandibulares

Hallazgo tomográfico^┼^	ATM derecha	ATM izquierda	Total
	n (%)	n (%)	n (%)
Condilar			
Aplanamiento condilar	10 (76,92%)	8 (61,54%)	18 (69,23%)
Erosión condilar	3 (23,08%)	4 (30,77%)	7 (26,92%)
Esclerosis subcondral	1 (7,69%)	2 (15,38%)	3 (11,54%)
Osteofito	1 (7,69%)	1 (7,69%)	2 (7,69%)
Quiste subcondral	0 (0,00%)	1 (7,69%)	1 (3,85%)
Espacio articular			
Disminución del espacio articular posterior	8 (61,54%)	4 (30,77%)	12 (46,15%)
Disminución del espacio articular superior	2 (15,38%)	2 (15,38%)	4 (15,38%)
Disminución del espacio articular anterior	1 (7,69%)	1 (7,69%)	2 (7,69%)
Disminución del espacio articular medial	0 (0,00%)	1 (7,69%)	1 (3,85%)

^┼^ = Un paciente pudo tener más de un hallazgo tomográfico.

Antes de la colocación de la FOE, la intensidad del dolor, evaluada mediante EVA, fue de 6,15 ± 1,82. Treinta días después del inicio del tratamiento, el dolor disminuyó significativamente a 3,62 ± 2,06 (p < 0,001). Al comparar los resultados según el sexo, la reducción fue significativa en mujeres (p < 0,001), mientras que en varones no se observaron cambios estadísticamente relevantes (p = 0,078) (Tabla 2).

**Tabla 2:** Comparación del dolor percibido (EVA basal y a los 30 días) en pacientes con trastornos temporomandibulares tratados con férula oclusal de estabilización, según el sexo

		EVA basal		EVA 30 días		Valor p
		Media	DE	Media	DE		
Sexo	Masculino	7,75	1,26	5,50	1,73	0,078a
	Femenino	5,44	1,59	2,78	1,64	0,003a*
	Total	6,15	1,82	3,62	2,06	<0,001a*

DE = Desviación estándar; a = Valor p obtenido de la prueba T de Student para muestras relacionadas; * = significancia estadística (p < 0,05)

**Figura 1:**
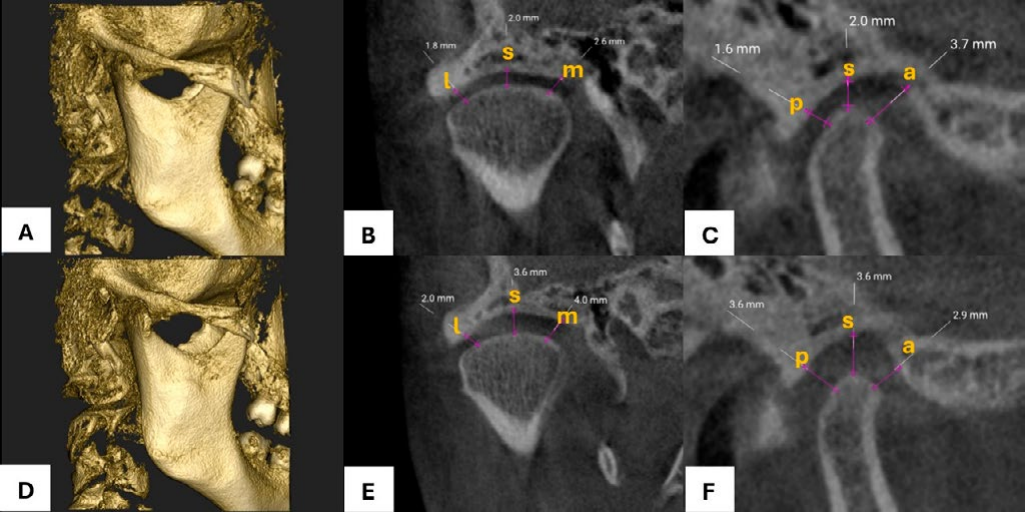
Tomografía computarizada de haz cónico del espacio articular temporomandibular en boca cerrada en pacientes con trastornos temporomandibulares. A-C) Imágenes obtenidas antes del tratamiento. D-F) Imágenes con la férula oclusal de estabilización. A y D) Reconstrucción tridimensional (3D). B y E) Corte coronal. C y F) Corte sagital. a: anterior; p: posterior; s: superior; m: medial; l: lateral

Se realizaron mediciones de los espacios de la ATM (Figura 1) y se compararon los promedios de los espacios anterior, superior, posterior, lateral y medial en ambos lados (derecho e izquierdo), antes y después del tratamiento. Todos los espacios articulares se midieron en milímetros (mm) y se expresaron como media ± desviación estándar (DE). Se observó una ligera disminución significativa en el espacio anterior derecho y un ligero aumento en los espacios anterior izquierdo, superior y medial derecho (p < 0,05). En cambio, el espacio posterior mostró un incremento aproximado de 1 mm en ambos lados, sin diferencia estadísticamente significativa (Tabla 3).

**Tabla 3:** Espacios de la ATM durante el pretratamiento y postratamiento con férula de estabilización oclusal, según lado

Espacio articular			Tratamiento				Valor p
			Pretratamiento		Postratamiento			
			Media	DE	Media	DE		
Espacio anterior	Lado	Derecho	2,14	0,74	1,92	0,64	<0,01a*
		Izquierdo	2,10	0,78	2,20	0,72	<0,01a*
Espacio superior	Lado	Derecho	2,05	0,66	2,72	0,88	<0,01a*
		Izquierdo	2,12	0,74	2,35	0,86	0,34a
Espacio posterior	Lado	Derecho	1,78	0,57	2,95	0,95	0,19a
		Izquierdo	1,88	0,43	2,74	0,80	0,70a
Espacio Lateral	Lado	Derecho	1,73	0,60	2,03	0,87	0,23a
		Izquierdo	1,80	0,65	1,97	0,65	0,32b
Espacio medial	Lado	Derecho	1,88	0,51	2,75	0,95	<0,01a*
		Izquierdo	2,38	0,94	2,76	0,79	0,15a

DE = Desviación estándar; a = Valor p obtenido de la prueba T de Student para muestras relacionadas; b = Valor p obtenido de la prueba de Wilcoxon; * = significancia estadística (p < 0,05)

## DISCUSIÓN

En esta serie de casos se evaluó a pacientes con TTM que presentaron dolor crónico, predominantemente mujeres (n = 9; 69,23%), lo que concuerda con lo reportado por Ortiz-Culca *et al*. ^18^ y Benites-Vega *et al*. ^17^ en población peruana. Tras 30 días de uso continuo de la FOE, se observó una reducción significativa en la intensidad del dolor percibido, hallazgo que coincide con la revisión sistemática de Hidalgo *et al*. ^6^, quienes evidenciaron la eficacia de la FOE en el control de la sintomatología de los TTM. Al comparar por sexo, la disminución del dolor fue significativa únicamente en mujeres. No obstante, la interpretación debe ser cautelosa debido al tamaño reducido de la muestra. 

Al respecto, se ha reportado que las mujeres con TTM presentan un umbral de dolor más bajo, caracterizado por mayor sensibilidad a la presión en la ATM, los músculos masticatorios y otras áreas corporales ^19^. La evidencia sugiere que los estrógenos pueden modular la percepción del dolor y la inflamación articular, lo que aumenta la sensibilidad femenina a los TTM y, por ende, la respuesta al tratamiento ^20^. Factores psicológicos, como mayor estrés y reactividad emocional al dolor, podrían amplificar la percepción de alivio en mujeres tras el tratamiento ^19^^,^^20^. Por ello, se recomienda que futuros estudios consideren muestras con participación equilibrada según sexo.

Sobre los hallazgos imagenológicos, los pacientes presentaron inicialmente un espacio articular anterior ligeramente mayor que el posterior. Tras la colocación de la FOE, se observaron modificaciones en los espacios articulares, con una disminución significativa del espacio anterior derecho y un aumento en los espacios anterior izquierdo, superior y medial del lado derecho. Asimismo, se registró un incremento aproximado de 1 mm no significativo en los espacios posteriores de ambos lados. Estos resultados son similares a los reportados por Musa *et al*. ^8^.

No obstante, Ahmed *et al*. ^7^ informaron aumentos significativos en los espacios posterior y superior específicamente en el lado desviado, mientras que Derwish y Pawlowska ^21^ no encontraron cambios significativos del tratamiento con FOE. Esta variabilidad podría explicarse por diferencias en el tipo de TTM y la presencia de desviaciones mandibulares, factores que deben considerarse en futuras investigaciones ^8^^,^^22^. Asimismo, la dispersión observada en las mediciones de los espacios articulares podría estar relacionada con el tamaño reducido de la muestra, lo que constituye una limitación del estudio.

Zhou *et al*. ^23^, Alhammadi *et al*. ^24^, Alhammadi *et al*. ^25^ y Aboalnagaa *et al*. ^26^ reportaron un mayor espacio articular anterior en pacientes con TTM, en comparación con individuos sanos. Asimismo, Yasa *et al*. ^27^ y Peroz *et al*. ^11^ asociaron un espacio posterior disminuido con una posición condilar mandibular más posterior y superior en pacientes con TTM. Por su parte, Lee *et al*. ^10^ informaron que un espacio articular anterior aumentado se asocia con síntomas clínicos de dolor y disfunción mandibular. Estas condiciones fueron similares a las mediciones identificadas en las tomografías de diagnóstico de los pacientes incluidos en la presente serie de casos. 

Desde una perspectiva funcional, se ha planteado que el uso de una FOE podría inducir una redistribución de las distancias cóndilo-fosa en la ATM ^9^^,^^21^. La personalización del diseño de la FOE favorecería una distribución más equilibrada de las cargas articulares y disminuiría así la concentración de estrés en la banda medial y posterior del disco articular, al desplazar las fuerzas hacia la zona intermedia ^28^. Este mecanismo ha sido propuesto como una posible explicación para la reducción del espacio anterior y el aumento no significativo del espacio posterior observados en esta serie de casos. Por tanto, la personalización de la férula constituye un componente esencial para lograr una adecuada estabilización oclusal ^29^. 

En el Perú no se han identificado estudios que evalúen los cambios inmediatos en los espacios articulares de la ATM tras la instalación de FOE. No obstante, Alvitez ^30^ reportó que el aumento controlado de la dimensión vertical oclusal mediante topes de silicona de 2 a 4 mm disminuyó el espacio articular anterior y aumentó el posterior. En esta serie de casos se observó un patrón biomecánico similar, lo que sugiere un desplazamiento condilar en la misma dirección. Por su parte, Dapello ^31^ analizó tomografías de pacientes peruanos sin sintomatología de TTM, pero con signos degenerativos condilares, y describieron un aplanamiento frecuente asociado a un espacio articular posterior ligeramente mayor que el anterior, posiblemente como respuesta adaptativa del cóndilo frente a cargas articulares. En el presente estudio, más de la mitad de los pacientes presentaron este signo acompañado de dolor articular, dolor muscular y disfunción mandibular, hallazgos compatibles con una fase activa del trastorno.

Otra limitación de este estudio fue el corto periodo de seguimiento, lo que exige interpretar los resultados con cautela. En tratamientos de tres meses o más podrían observarse modificaciones en la cortical ósea y la posición condilar, mientras que entre seis y doce meses podrían producirse cambios dentoalveolares adversos asociados con el uso prolongado de la FOE ^7^^,^^8^^,^^12^. Estos aspectos deberían ser abordados en futuras investigaciones. Además, se ha informado que, tras la suspensión de la férula, pueden presentarse recurrencias del dolor o de la disfunción articular ^32^. Por ende, el seguimiento clínico minucioso por parte del especialista resulta fundamental durante el tratamiento de los TTM.

Este estudio corresponde a la primera etapa del manejo terapéutico; en fases posteriores, se prevé realizar ajustes periódicos de la FOE y, en algunos casos, infiltraciones de anestésico local sin vasoconstrictor para el control del dolor muscular. Por ello, el seguimiento individualizado es esencial para valorar la evolución clínica posterior al uso de la FOE. En conjunto, los resultados constituyen un punto de partida relevante para futuros estudios que evalúen no solo el manejo con FOE, sino su complementación con otras alternativas terapéuticas.

## CONCLUSIÓN

El uso de la FOE en pacientes con TTM mostró una reducción significativa en la intensidad del dolor y produjo modificaciones inmediatas en los espacios articulares. Estos resultados respaldan la eficacia terapéutica de la FOE en el control del dolor y en la función articular. Sin embargo, se requieren estudios con muestras más amplias y un seguimiento prolongado que permitan evaluar los cambios estructurales y funcionales a largo plazo, así como la posible influencia del sexo en la respuesta clínica al tratamiento.

## Fuente de financiamiento:

Autofinanciado
